# Supplementary figures and images for: P2 Receptors Influence hMSCs Differentiation towards Endothelial Cell and Smooth Muscle Cell Lineages
Source: Int J Mol Sci. 2020 Aug 27;21(17):6210. doi: 10.3390/ijms21176210 (PMC7503934; doi:10.3390/ijms21176210)

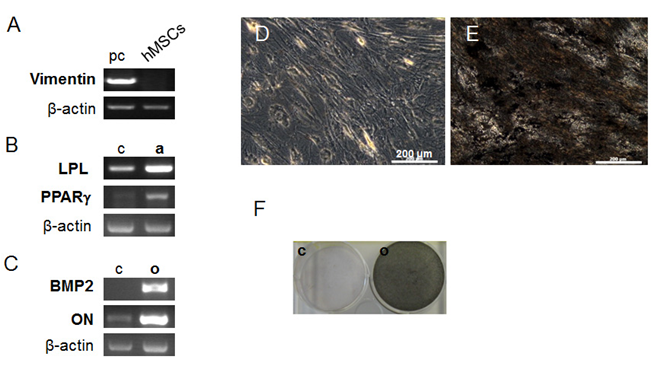

Supplement: Supplementary file 1 [file ijms-21-06210-s001.zip › Supp Material/Figure S1.TIF]

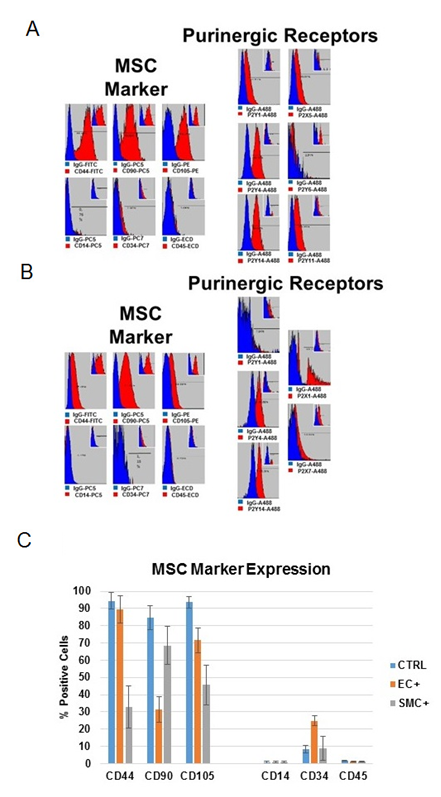

Supplement: Supplementary file 1 [file ijms-21-06210-s001.zip › Supp Material/Figure S2.TIF]

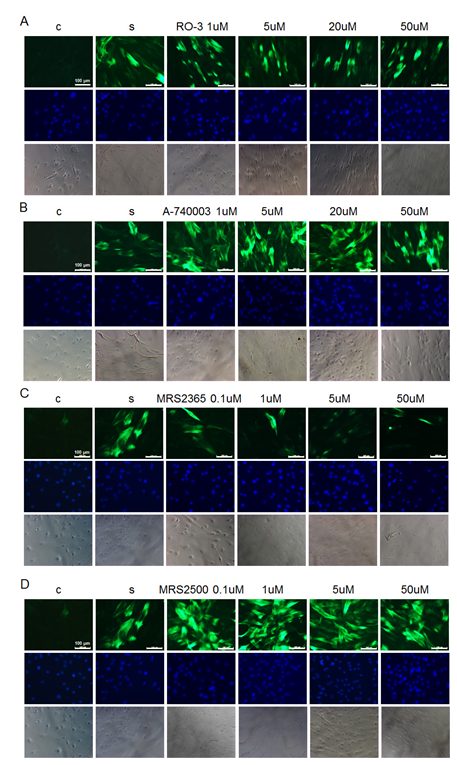

Supplement: Supplementary file 1 [file ijms-21-06210-s001.zip › Supp Material/Figure S3.TIF]

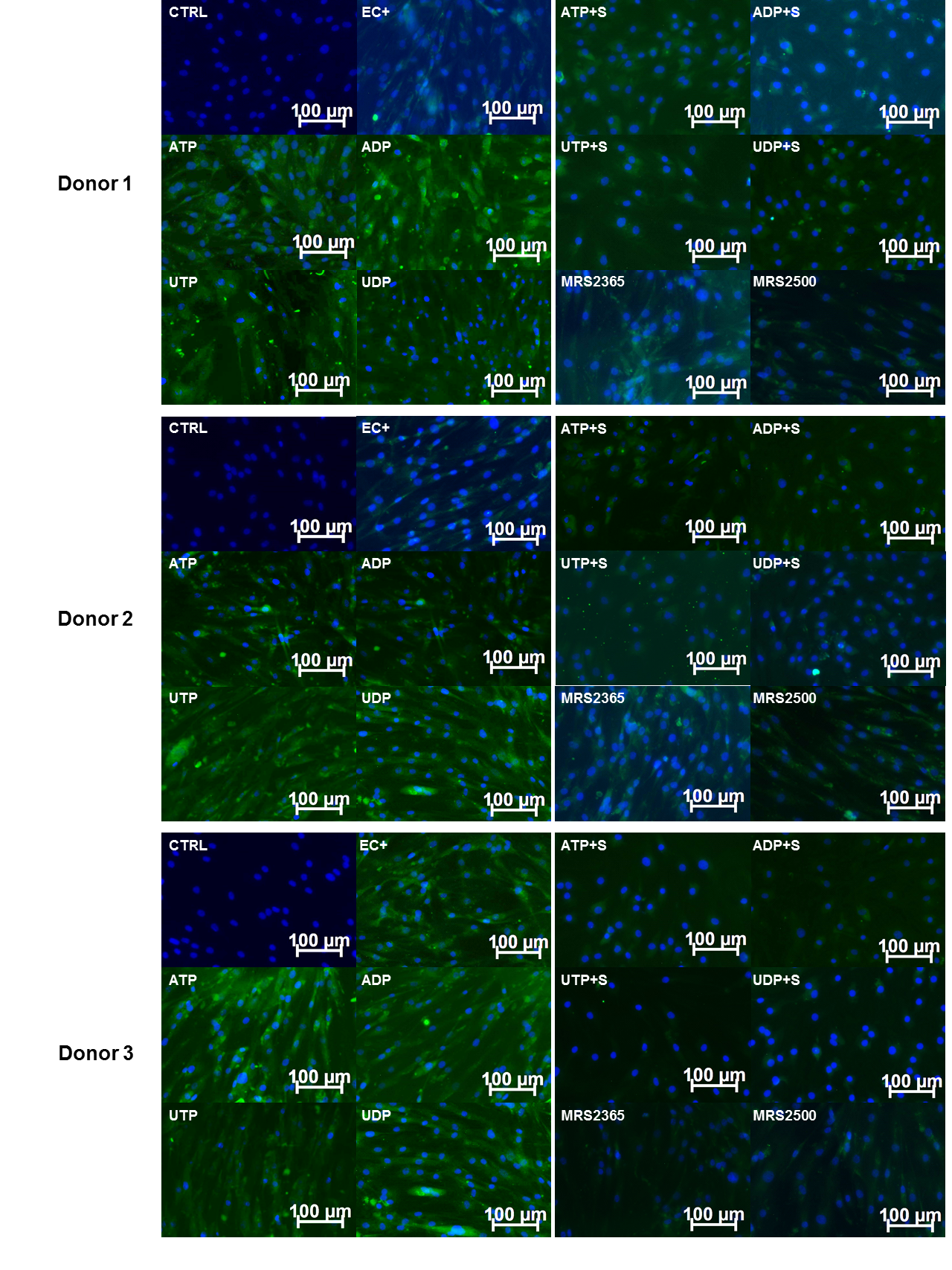

Supplement: Supplementary file 1 [file ijms-21-06210-s001.zip › Supp Material/Figure S4.TIF]

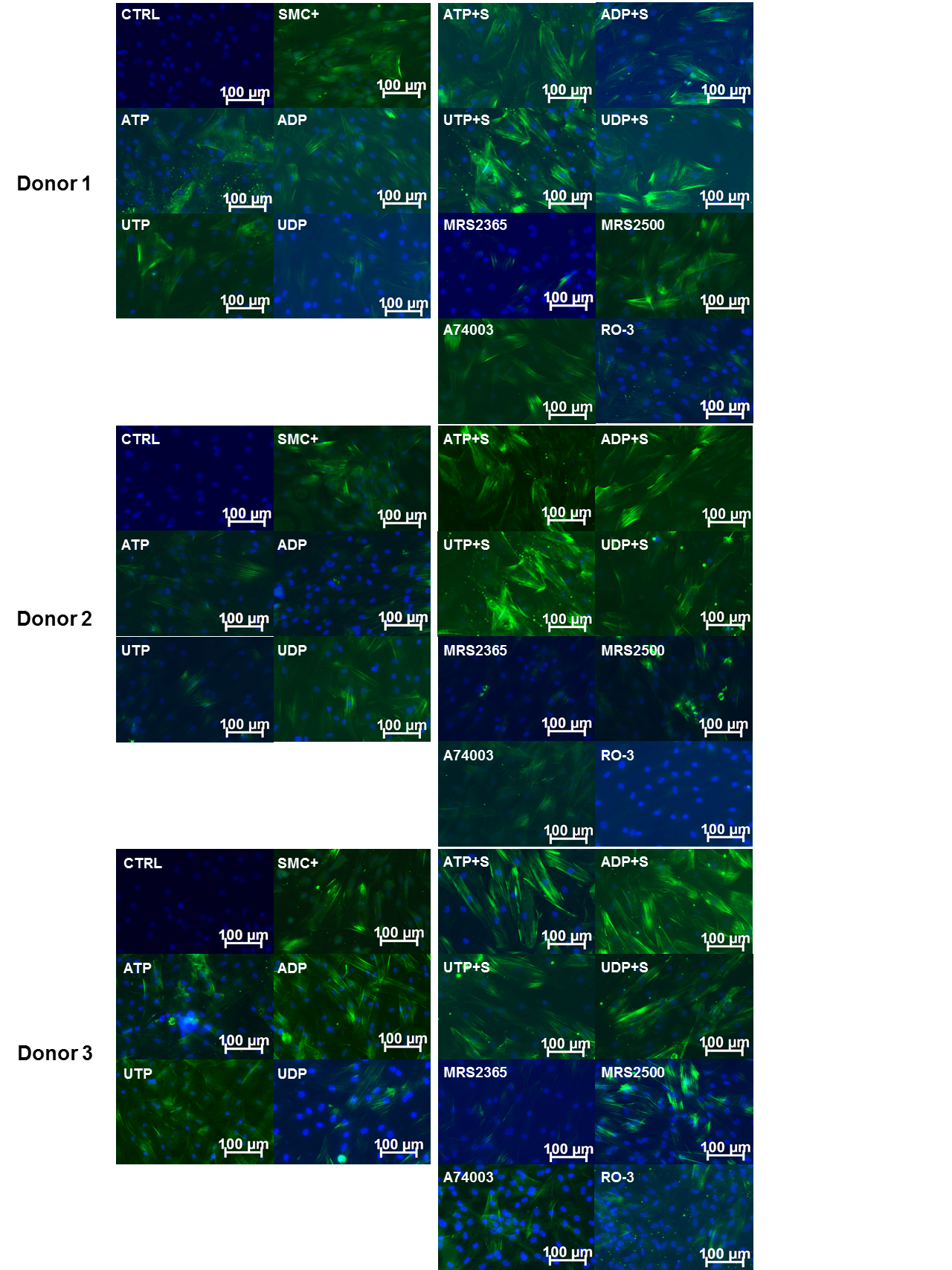

Supplement: Supplementary file 1 [file ijms-21-06210-s001.zip › Supp Material/Figure S5.TIF]

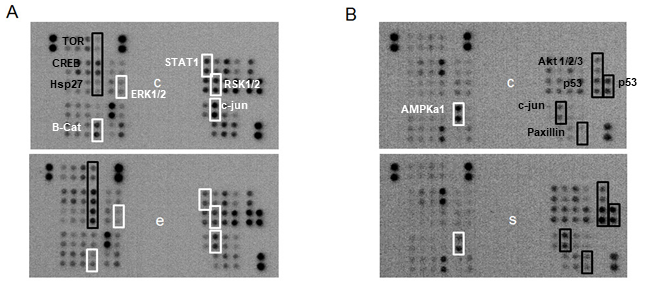

Supplement: Supplementary file 1 [file ijms-21-06210-s001.zip › Supp Material/Figure S6.tif]

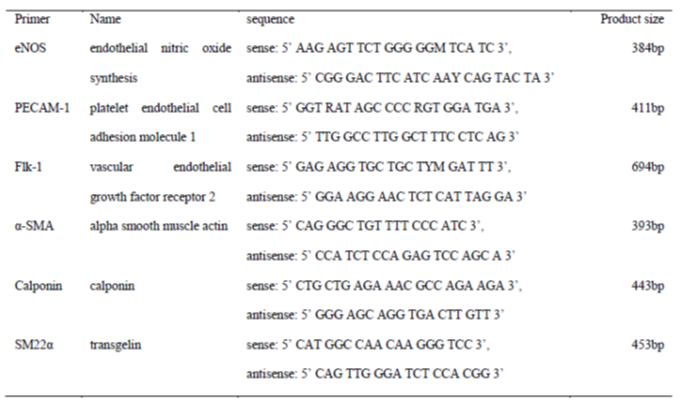

Supplement: Supplementary file 1 [file ijms-21-06210-s001.zip › Supp Material/Table S1.TIF]
